# The Spiritual Rappers Once More

**Published:** 1852-07

**Authors:** 


THE SPIRITUAL RAPPERS ONCE MORE.

Our readers will remember the exposure made of the "Spiritual Rappers," by Dr. Lee, Dr. Flint, and others, at Buffalo, a few months since. Notwithstanding that the pretensions of these young ladies were so effectually exposed on that occasion, they are still perambulating the country, imposing upon the credulous, and turning the heads of a few weak people. They paid a visit to Kentucky, a short time since, and were in Lexington, where they were met by our friend, Dr. Darby. We have received from the doctor a long and amusing account of his interview with the *Misses Fox*, the sum of which is, that when they found he had come to investigate rather than to stare, they refused to perform. We would give the whole of his interesting letter if we did not deem the humbug unworthy of any further notice. Suffice it to say, the Misses Fox made a very short stay in Lexington. They felt that their tricks had been exposed, for, Dr. Darby adds, "they would not appear the day after he saw them although they remained in the city till the afternoon train of cars left for Louisville." We learn from Dr. D., that more than one physician had marvelous stories to tell of the fair jugglers, after an interview with them, and no doubt that wherever the "Rappers" go they will find, even among the learned, good subjects to help on with the imposture. Nevertheless, we think it must now soon come to an end.

